# Climate Driven Egg and Hatchling Mortality Threatens Survival of Eastern Pacific Leatherback Turtles

**DOI:** 10.1371/journal.pone.0037602

**Published:** 2012-05-23

**Authors:** Pilar Santidrián Tomillo, Vincent S. Saba, Gabriela S. Blanco, Charles A. Stock, Frank V. Paladino, James R. Spotila

**Affiliations:** 1 Department of Biology, Drexel University, Philadelphia, Pennsylvania, United States of America; 2 The Leatherback Trust, Goldring Marine Biology Station, Playa Grande, Costa Rica; 3 Atmospheric and Oceanic Sciences Program, Princeton University, Princeton, New Jersey, United States of America; 4 NOAA, Geophysical Fluid Dynamics Laboratory, Princeton, New Jersey, United States of America; 5 Department of Biology, Indiana-Purdue University, Fort Wayne, Indiana, United States of America; University of Canterbury, New Zealand

## Abstract

Egg-burying reptiles need relatively stable temperature and humidity in the substrate surrounding their eggs for successful development and hatchling emergence. Here we show that egg and hatchling mortality of leatherback turtles (*Dermochelys coriacea*) in northwest Costa Rica were affected by climatic variability (precipitation and air temperature) driven by the El Niño Southern Oscillation (ENSO). Drier and warmer conditions associated with El Niño increased egg and hatchling mortality. The fourth assessment report of the Intergovernmental Panel on Climate Change (IPCC) projects a warming and drying in Central America and other regions of the World, under the SRES A2 development scenario. Using projections from an ensemble of global climate models contributed to the IPCC report, we project that egg and hatchling survival will rapidly decline in the region over the next 100 years by ∼50–60%, due to warming and drying in northwestern Costa Rica, threatening the survival of leatherback turtles. Warming and drying trends may also threaten the survival of sea turtles in other areas affected by similar climate changes.

## Introduction

Reproductive success of egg-burying species such as sea turtles depends upon the stability of the nest environment [1], [2]. For example, it is suggested that crocodilians and turtles survived the mass extinction at the end of the Cretaceous Period in part because they laid eggs in substrates that provided relatively stable temperature and humidity throughout incubation [3], [4]. Variations in temperature and humidity occur in the underground nest environment and are greater in freshwater turtle and lizard nests that are shallower than sea turtle nests [5]. Water exchange and incubation temperature influence hatching success of eggs and fitness of hatchlings. High temperature and dry substrates are unfavorable for egg development and hatchling quality [2], [5]–[8] and can cause the loss of an entire reproductive season [9]. Thus, changes in local climate can have important effects on reproductive success of reptiles.

El Niño Southern Oscillation (ENSO) has profound effects on many ecological processes [10], [11]. It alternates from “El Niño” periods when sea surface temperature (SST) in the eastern tropical Pacific is warmer than normal, to “La Niña” when SST is cooler than normal, both of variable duration and strength, with ENSO neutral conditions in between. ENSO affects local weather in diverse ways. Warm El Niño events are associated with increased precipitation in the Galápagos Islands [12], [13] and floods in South America, whereas they are associated with drought in Australia and Indonesia [14].

Leatherback turtles (*Dermochelys coriacea*) nest on tropical and subtropical beaches around the world, but are critically in danger of extinction [15] due to anthropogenic impacts such as egg poaching and bycatch in industrial and artisanal fisheries [16], [17], [18]. Populations of leatherback turtles have been greatly reduced in the eastern Pacific and Playa Grande, in northwestern Costa Rica remains one of the last major nesting sites in the region [19], [20]. Female leatherbacks in the eastern Pacific nest on average 7 times in a season and return to the nesting beach every 3.7 years [20], [21]. This remigration interval can be prolonged during El Niño conditions or reduced during La Niña conditions [22], [23]. Eggs are buried ∼80 cm deep in the sand, incubate for ∼60 days [24] and, after hatching, hatchlings emerge synchronously from the nest [25]. Temperature of the sand affects the ability of hatchlings to emerge from the nest [26]. Furthermore, leatherback turtles, as well as other reptiles, have temperature-dependent sex determination (TSD) and El Niño causes a female-biased sex ratio [24], [27]. Climate change may threaten survival of leatherback populations even if other factors driving population declines are removed.

**Figure 1:**
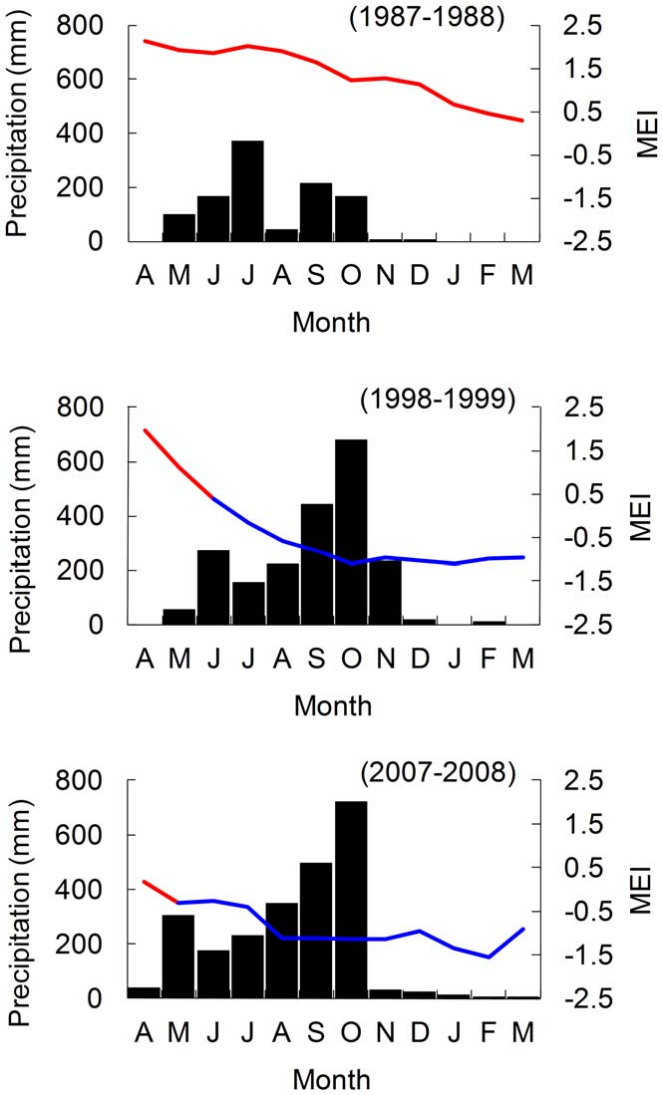
Monthly distribution of precipitation in Liberia, Costa Rica. Precipitation is highly variable between years of El Niño (1987–1988) and La Niña (1998–1999, 2007–2008). The Multivariate ENSO Index (MEI; solid line) indicates when the Pacific climate is in El Niño (red) and La Niña (blue) phases [34].

Monthly precipitation in North Pacific Costa Rica is influenced by ENSO. Rainy seasons during El Niño events occur but levels of rain are low, causing droughts during the following dry season [28]. The rainy season extends from May to November with September and October being the rainiest months, followed by the dry season from December to April (Fig. 1). The leatherback nesting season starts in October and ends at the end of February. Because the incubation period lasts two months, many clutches are still incubating during the peak of the dry season when sand temperature is maximal, which reduces hatching success and emergence of hatchlings from the nest [7], [9], [26].

The objective of this study was to test the effects of climatic conditions on the incubation of leatherback eggs and emergence of hatchlings from the nest. We found that hatching success of eggs and emergence rate of hatchlings from the nest were strongly affected by the prevailing climatic conditions. Our projections showed that hatchling output will decline throughout the 21^st^ century under projections of climate change.

## Materials and Methods

We conducted the study at Playa Grande, Parque Nacional Marino Las Baulas (10°20 N, 85°51 W), located on the North Pacific coast of Costa Rica. We included six nesting seasons in the analyses, from 2004–2005 to 2009–2010. Each season included clutches laid from October of each year to January of the next. Clutches laid in February were removed from the analyses because of low sample sizes. We marked nests during egg oviposition and excavated them two days after hatchlings emerged. In some instances we could not mark them because we missed the turtle, we found her when she was already covering the nest, or we lost the nest because of inaccurate triangulations. We excavated and included a total of 814 nests in the analyses (31% of the total estimated number of clutches laid between 2004–2005 and 2009–2010) from 283 females (70% of the females) (Table 1).

**Table 1:** Number of female turtles and nests registered per season and included in the analyses.

	Seasons					
	2004–2005	2005–2006	2006–2007	2007–2008	2008–2009	2009–2010
Total number of turtles	54	124	76	81	27	41
Number of turtles represented	40	71	44	65	26	37
Total number of nests	358	772	536	507	170	258
Number of nests analyzed	128	172	130	158	99	127

Total number of nests was obtained multiplying the total number of females identified in a season by the estimated clutch frequency for that year, following the methodology described in [45].

We estimated hatching success as the percentage of eggs within a clutch that completed development, and emergence rate as the percentage of hatchlings that successfully emerged from the nest within two nights of the initial emergence event. We used the formula *H* = *S*/(*S*+*U*) to estimate hatching success (*H*), where *S* was number of empty eggshells (more than 50% of the shell remained [29]) and *U* was the number of unhatched eggs as previously described [26]. We used the formula *R* = (*S*−(*L*+*D*))/*S* to estimate emergence rate (*R*), where *L* and *D* were live and dead hatchlings found in the nest respectively at excavation time. We estimated mean monthly hatching success and mean monthly emergence rate by month eggs were laid (October - January).

We obtained local climate data (monthly precipitation and air temperature) from a weather station located at the Daniel Oduber Quirós International Airport in Liberia, Costa Rica (approximately 50 km from study site). Through a regression analysis, we determined the effects of ambient temperature during the two incubating months and precipitation accumulated over different periods of time (during the two months of incubation, one, two and three months before eggs were laid and total precipitation during rainy season), on both hatching success and emergence rate. We then used the regression equations from the analyses of precipitation and ambient temperature that gave the strongest signal on monthly hatching success and emergence rate to produce a hindcast of mean annual estimates from 1976 to 2010, and average annual values considering the temporal distribution of nests (on average 13%, 27%, 35% and 25% of clutches are laid in October, November, December and January respectively). Finally, we compared the hindcast of hatching success and emergence rate to the September - October Multivariate ENSO Index (MEI) for the same time period. This index serves as an indicator of the strength of the El Niño and La Niña events. The MEI is calculated bimonthly and based on six observed variables over the tropical Pacific: sea level pressure, surface zonal and meridional wind components, sea surface temperature, surface air temperature, and cloudiness [30]. The MEI data were available at http://www.esrl.noaa.gov/psd/people/klaus.wolter/MEI/table.html.

### Projections from Global Climate Models Throughout the 21^st^ Century

We used climate model projections contributed to the Intergovernmental Panel on Climate Change (IPCC) fourth assessment report (AR4) to assess the potential effects of climate change on egg development and hatchling emergence at Playa Grande up to the year 2100 under the SRES A2 development scenario [31]. We used the regression equations for hatching success and emergence rate based on the observed response to climate variability over the last decade, together with individual climate models and the ensemble mean projected monthly values of precipitation and air temperature for the area encompassing the northwest coast of Costa Rica.

We used bias-corrected monthly precipitation and ambient temperature projections from the ensemble of IPCC models. The ensemble (17 models) data were extracted from the World Climate Research Programme’s Coupled Model Intercomparison Project phase 3 (CMIP3) multi-model dataset [32] under the IPCC SRES A2 greenhouse gas emissions scenario. We bias-corrected each CMIP3 model’s projection based on observed climate data for the period of 1976 to 2000. For bias-correction, we extracted historical runs (Climate of the 20th century experiment) for each CMIP3 model for the same time period as the observed data in Liberia. For each month, we calculated a bias-correction factor by dividing the mean observed data by the mean modeled data. We used equations from the empirical analysis on hatching success and emergence rate together with each individual model’s projected precipitation and air temperature (bias-corrected) for the area encompassing northwest Costa Rica.

To more closely assess how projected changes in ENSO variability and mean warming and drying trends interact to determine hatching success and emergence rate, we examined projections from a subset of the climate model ensemble (*n* = 5) that most skillfully captured present day ENSO characteristics and feedbacks [33].

Of the six years, nesting seasons 2004–2005, 2006–2007 and 2009–2010 took place during El Niño conditions and seasons 2005–2006, 2007–2008 and 2008–2009 during La Niña conditions. The El Niño in 2009–2010 and the La Niña in 2007–2008 were the strongest of the six events [34].

## Results

Mean (±SD) annual hatching success and mean (±SD) annual emergence rate of leatherback clutches at Playa Grande for 2004–2005 to 2009–2010 were 0.47±0.25 and 0.82±0.22 respectively.

**Figure 2:**
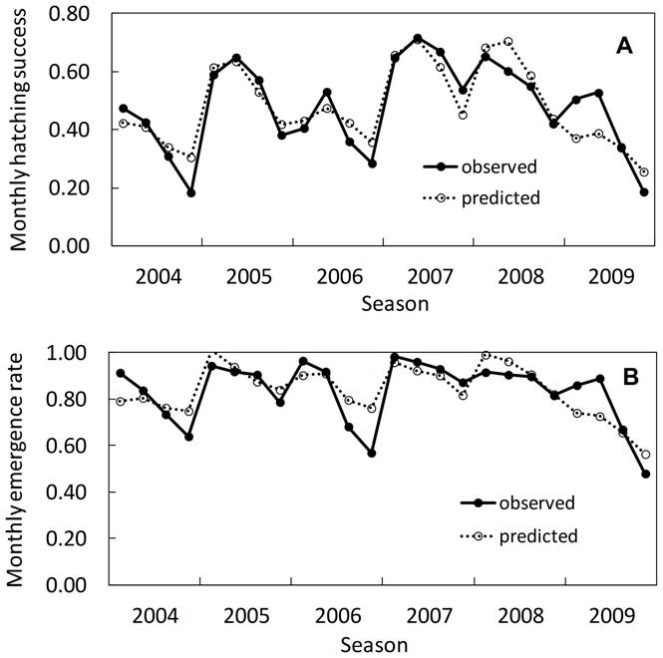
Hatching success and emergence rate of leatherback nests at Playa Grande, between 2004–2005 and 2009–2010. (a) Observed versus predicted mean monthly hatching success. Prediction was based on local weather conditions (*y = a+bx_1_+cx_2_−dx_3_*; *R^2^* = 0.81, *P*<0.001); where *x_1,_ x_2_* and *x_3_* were precipitation accumulated in the two previous months, precipitation in October and mean ambient temperature during incubation respectively. Regression coefficients (± SE) were *a = *1.87±0.76, *P*<0.05; *b* = 1.83×10^−4^±4.94×10^−5^, *P*<0.002; *c* = 3.23×10^−4^±8.02×10^−5^, *P* = 0.001; *d* = −6.06×10^−2^±2.79×10^−2^, *P*<0.05. (b) Observed versus predicted mean monthly emergence rate. Prediction was based on local weather (*y = a−bx_1_+*c*x_2_*; *R^2^* = 0.65, *P*<0.001); where *x_1_* and *x_2_* were mean ambient temperature and precipitation accumulated in September-October respectively. Regression coefficients (± SE) were *a = *4.51±0.81, *P*<0.001; *b* = –0.14±0.03, *P*<0.001; *c* = 1.62×10^−4^±4.80×10^−5^, *P*<0.01.



Climate variability influenced hatching success and emergence rate of leatherback clutches. Low precipitation and high temperatures were detrimental to both eggs during development and hatchlings during emergence (Fig. 2). Mean monthly hatching success was influenced by precipitation accumulated in the two months before eggs were laid, precipitation in October (immediately prior to the onset of the dry season), and average ambient temperature during the two months of incubation, The relationship was defined by a stepwise multiple regression equation:

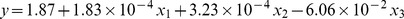



where *x_1_* = precipitation accumulated in the two months before eggs were laid, *x_2_* = precipitation in October and *x_3_* = mean ambient temperature during the two months of incubation (*R^2^* = 0.81, *n* = 24, *P*<0.001, Fig. 2A). The standardized partial regression coefficients were 0.46, 0.45 and - 0.24 respectively. Therefore, precipitation in the two previous months, precipitation in October and ambient temperature during incubation explained 40, 39 and 21% respectively of the hatching success variation accounted for by the model.

Monthly emergence rate was best explained by the average ambient temperature during the two months of incubation and the total precipitation in September and October. The relationship was defined by a stepwise multiple regression equation:
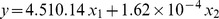

*x_1_* = mean ambient temperature during the incubation months and *x_2_* = precipitation accumulated in September – October (*R^2^* = 0.65, *n* = 24, *P*<0.001, Fig. 2B). The standardized partial regression coefficients were - 0.62 and 0.44 respectively. Thus, air temperature during incubation and total precipitation in September and October explained 58 and 42% respectively of the variation in emergence rate explained by the model. Therefore, precipitation and ambient temperatures, characterized by a strong interannual variability, affected (1) eggs during development and (2) hatchlings during emergence through the ∼80 cm sand column.

**Figure 3:**
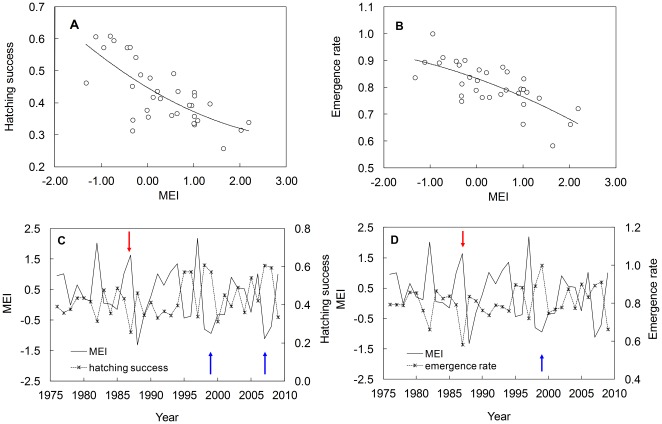
Hatching success and emergence rate hindcasts from 1976–1977 to 2009–2010 related to MEI. (a,b) hindcast of hatching success and emergence rate in relation to MEI of September-October (*R^2^* = 0.52, *P*<0.001 for both); (c,d) hindcast of hatching success and emergence rate (dashed lines with x’s) related to MEI (solid lines) of September-October by year. Lowest and highest hindcasts are marked with red and blue arrows respectively.

We found that MEI was significantly correlated with hatching success (quadratic equation: y = 0.45–0.09 *x*+0.01 *x^2^*, *R^2^* = 0.52, *n* = 34, *P*<0.001) and emergence rate (quadratic equation: y = 0.83–0.06 *x* −0.01 *x^2^*, *R^2^* = 0.52, *n* = 34, *P*<0.001), with positive values of MEI (El Niño) corresponding to years of low hatching success and emergence rates and negative values of MEI (La Niña) corresponding to years of high hatching success and emergence rates (Fig. 3A,B). Our regression model estimated the lowest hatching success and emergence rate for 1987, a year of strong El Niño conditions, while estimating the highest hatching success for 1998 and 2007, and highest emergence rate for 1999, all during La Niña events (Fig. 3C,D).

### Projections from Global Climate Models Throughout the 21^st^ Century

**Figure 4:**
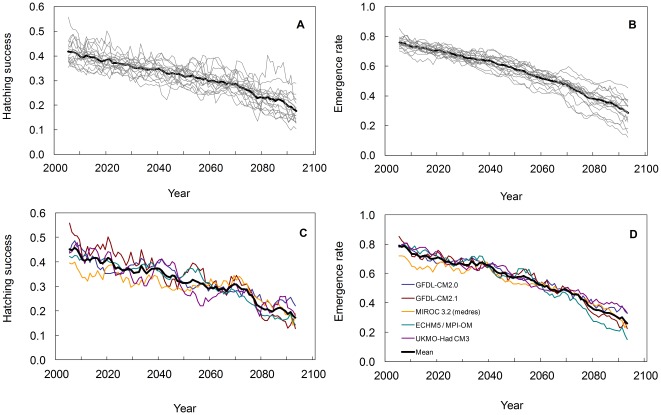
Hatching success and emergence rate projections of leatherback nests in 100 years of climate change. Projections are based on (a,b) each of the 17 bias-corrected CMIP3 models (grey lines) and the ensemble mean (black line) and (c,d) 5 of the bias-corrected CMIP3 models that are skilful at resolving present-day ENSO variability (coloured lines) and their ensemble mean (black line). Time-series projections are presented as 10-year moving averages.

Our model projected that both hatching success (Pearson correlation, *r* = −0.91, p<0.01) and emergence rate (Pearson correlation, *r* = −0.97, p<0.01) would significantly decrease between years 2001 and 2100 due to a warming and drying of the area encompassing northwest Costa Rica (Fig. 4). Of the 17 IPCC models used here, 13 of them projected a decrease in precipitation while all models projected an increase in air temperature. Our projections indicated that hatching success would decrease from a 10-year moving average ∼0.42 to ∼0.18 from the beginning to the end of the 21^st^ century, and emergence rate from ∼0.76 to ∼0.29 (Fig. 4A,B).

The subset of models that best captured ENSO variability produced projections of hatching success and emergence rate that had similarly decreasing trends to those of the full ensemble (Fig. 4C,D) despite diverging in their projections of changes in ENSO variability over the next 100 years [33]. Here, projected hatching success decreased from ∼0.45 to ∼0.17 and emergence rate from ∼0.79 to ∼0.26 (Fig. 4C,D).

## Discussion

Local climate had a strong effect on leatherback eggs and on hatchlings that must dig through 80 cm of sand. Dry and warm conditions at Playa Grande were harmful to both eggs and hatchlings. High temperatures during incubation also create lethal conditions for olive ridley turtle (*Lepidochelys olivacea*) eggs in Pacific Costa Rica during the dry season [9] and decrease emergence rate of green turtles (*Chelonia mydas*) in Caribbean Costa Rica [35].

Precipitation creates suitable conditions of humidity inside the nest and cool temperatures favor development and prevent sand desiccation in this region. However, higher levels of precipitation can result in increased mortality of eggs or hatchlings as seen at other locations [36]. The strong interannual variability of rainfall at Playa Grande results in “boom-and-bust” cycles of recruitment in the population. Therefore, cool and pluvial La Niña years are extremely important for periods of enhanced leatherback neonate recruitment. Additionally, because leatherbacks exhibit temperature-dependent sex determination and air temperatures during La Niña events are cooler, these events are critical for the production of male hatchlings [37]. Within a season, during both El Niño and La Niña events, wet and cool conditions at the beginning of the nesting season result in a higher production of hatchlings than at the end of the season when warm and dry conditions persist [26]. Leatherbacks at Playa Grande typically nest in the open beach away from the vegetation and do not select for different nest sites along the sea to vegetation axis [38] and there is no shade available along the beach. Sea turtles in this type of tropical breeding site will have a lower fitness if they nest during the months when high temperatures would be detrimental for eggs and hatchlings. However, some nesting still occurs during the off season. Likewise during El Niño years, turtles still nest but in lower than average numbers. Historically, it is possible that some turtles benefited from low nest density found on the beach at these times, before declines started.

Hatching success and emergence rate, however, are influenced by variations in nest microclimate that are not fully captured by the MEI or the coarse monthly precipitation and temperature averages examined herein. For example, high temperatures inhibit emergence of sea turtle hatchlings [7] and high temperatures in the period just before emergence are associated with lower emergence rate [26], [35]. An example of where such dependence meteorological variations not captured by the metrics considered herein may have played a role is the 1997 El Niño. Despite being the strongest ENSO event recorded, the 1997 El Niño resulted in low annual hatching success and emergence rates but not lower than in 1987. This difference may be explained by the shorter time-scale meteorological variations or higher levels of rain accumulated in September and October of 1997 (271 and 182 mm) compared to 1987 (213 mm and 167 mm). Likewise, the 1988 La Niña was a very strong event but resulted in lower hatching success than in 1998 and 2007, when higher levels of rain were registered. Further exploration of the impact of meteorological variations over finer temporal and spatial scales than those captured by the metrics considered, may explain more variance in hatching and emergence success. However, the performance of the coarse-scale meteorological forcing considered herein as predictors (e.g., *R^2^* = 0.81 and *R^2^* = 0.65 for hatching and emergence respectively) suggests that they provide useful, robust indicators of climate effects. The utility of monthly precipitation and temperature metrics as predictors of hatching and emergence rates also facilitates integration with climate projection, which are archived as monthly outputs.

### Projections from Global Climate Models Throughout the 21^st^ Century

Most of the 17 projections contributed to the fourth assessment report of the IPCC projected a decrease in precipitation and all 17 models projected an increase in air temperature throughout the 21^st^ century in northwestern Costa Rica. These results are consistent with prior studies that also report a decrease in IPCC model precipitation over Central America over the year 2100 [39], [40]. If the nesting sites of sea turtle species in Central America become warmer and dryer, we can expect similar declines in hatching success and hatchling emergence of other species. Additionally, sex ratios at Playa Grande are already female-biased (∼90%) [37]. Therefore, increased ambient temperature from climate change may result in a minimal per-decadal production of male hatchlings in the region.

Our projections show a sharp decline from ∼42% to ∼18% in hatching success and from ∼76% to ∼29% in emergence rate between 2001 and 2100. These results are also robust to uncertainties in the regression analysis and are strongly supported by physiological studies [1], [2], [5], [6].

“Boom-and-bust” cycles of recruitment can still be expected under future climatic scenarios. However, our model suggests that the overall leatherback recruitment rate may become too low to maintain populations because of the rapid changes in climatic conditions. Leatherback turtles reach sexual maturity in ∼15 years [41] although recent studies suggest that it may take them up to 30 years to mature [42]. Other sea turtle species reach sexual maturity in ∼25**–**40 years [43], [44]. Therefore, the long generation times exhibited by sea turtles will make it very difficult for them to adapt to new environmental conditions caused by climate change.

Leatherback turtles at Playa Grande are currently endangered due to the impacts of fisheries, poaching, pollution, and tourist development on the nesting beaches. Our results show that even if these threats are eradicated, those turtles will still be threatened by climate change. Even considering human intervention at nesting beaches (i.e. irrigating nests, laboratory egg incubation, climate-controlled hatcheries), climate change may still become a major driver of the survival of leatherbacks due to the warming of Central America. Given the limited number of leatherback nesting beaches in Central America, which is governed by a variety of factors (i.e. tidal range, sand type, currents, predation, vegetation, beach development, etc.), the probability of a new nesting beach emerging in a cooler, wetter area throughout this century is unlikely. Similar drying and warming is projected throughout much of Central America by the majority of IPCC models [40]. Significant southward movement into the equatorial convergence zone would yield wetter but similarly warm conditions with altered rainfall seasonality. Significant northward movement into the sub-tropics would yield cooler but much drier conditions [Saba et al. in review]. Thus, leatherback turtles that live in the east Pacific and nest in Central America will face increasing environmental pressures on their survival if similar trends continue in the 21^st^ century. Local mitigation may not compensate for a lack of global action in reversing the trends. Other sea turtle species in Central America and in other drying and warming areas, whose egg and hatchling survival depend upon wetter, cooler climatic conditions, could follow a similar projected path as the leatherback turtle.
